# Patient-Reported Outcome Measures Assessing Genital Self-Image: A Systematic Review of Measurement Properties

**DOI:** 10.1007/s10508-026-03488-0

**Published:** 2026-07-13

**Authors:** Erisvan Vieira da Silva, Guilherme Tavares de Arruda, Leticia Amaral Corrêa, Melissa Medeiros Braz, Hedioneia Maria Foletto Pivetta

**Affiliations:** 1 Postgraduate Program in Movement Sciences and Rehabilitation, Federal University of Santa Maria, Santa Maria, Brazil; 2 Postgraduate Program in Physiotherapy, Federal University of São Carlos, São Carlos, Brazil; 3 Department of Chiropractic, Faculty of Medicine, Health and Human Sciences, Macquarie University, Sydney, Australia; 4 Department of Physiotherapy and Rehabilitation, Federal University of Santa Maria, Avenida Roraima, 1000, Camobi, Santa Maria, RS 97105-900 Brazil

**Keywords:** Genital self-image, Body image, PROM, COSMIN, GRADE

## Abstract

This systematic review synthesizes and critically appraises the measurement properties of genital self-image measures. Nine measures that met the definition of body image assessment (i.e., subjective perceptions related to the genitals) were included. These measures were the Female Genital Self-Image Scale (FGSIS), Genital Self-Image Scale, Genital Appearance Satisfaction, Male Genital Self-Image Scale, FGSIS Spanish version, Index of Male Genital Image, and Korean Genital Image Scale. Articles evaluating the measurement properties of these scales (*N* = 22) were assessed for methodological quality using the COSMIN Risk of Bias Checklist. The COSMIN criteria were used to summarize and classify the measurement properties of each PROM. The Grading of Recommendations Assessment, Development, and Evaluation graded the evidence. None of the PROMs had all of the measurement properties graded, and there was insufficient evidence to support the recommendations. Only the FGSIS 7-item version was able to assess the quality of evidence for content validity, and its use was potentially recommended. The included studies had insufficient measurement properties. Future studies should collect additional data to assess the measurement properties of the FGSIS 7-item version using a robust methodology based on guidelines for conducting studies on measurement properties of PROMs. The measurement properties of each PROM are discussed in detail, including aspects of interpretability and feasibility.

**Supplementary Information:**

The online version contains supplementary material available at 10.1007/s10508-026-03488-0.

## Introduction

### Relationship Between Body Image and Genital Self-Image

Body image is a central concept in understanding how individuals perceive and relate to their bodies (Kling et al., 2019). It involves the personal evaluation of physical characteristics and is closely linked to emotional and psychological aspects that directly influence self-esteem and body satisfaction (da Silva et al., 2024a, 2024b). Within this broad scope of perception, genital self-image (GSI) is a specific subset related to how individuals perceive and evaluate their own genitalia (Berman et al., 2003). Similar to body image, GSI is formed by sociocultural factors, personal and social experiences, and psychological influences (da Silva et al., 2024a, 2024b).

As an important component of body image, GSI has direct implications for sexual health and overall well-being (Gaither et al., 2017; Komarnicky et al., 2019; da Silva et al., 2023). However, despite the increasing interest in understanding body image and, by extension, GSI, the literature lacks a clear systematization of available patient-reported outcome measures (PROMs) for this assessment. This gap makes it difficult to select appropriate methods for both research and clinical practice. Therefore, a systematic review of GSI measurement instruments is needed to synthesize the advances in the field and provide recommendations based on the psychometric properties of these tools (Kling et al., 2019).

Although PROMs that assess GSI have been used more frequently in research, their clinical applicability is relevant. For example, Applebaum and Placik (2022) demonstrated that dissatisfaction with genital appearance significantly influences decisions to undergo cosmetic genital surgery. Furthermore, studies involving women with conditions such as inflammatory vulvar dermatoses and pelvic organ prolapse have demonstrated a clear association between genital dissatisfaction, reduced quality of life, and impaired sexual function (Djusad et al., 2021; Rivera et al., 2022). These findings suggest that GSI measures can be used to assess GSI in both research and clinical settings, identifying subjective distress that can guide decision-making and intervention planning. Additionally, although PROMs are often applied to evaluate treatment outcomes, they encompass any subjective report of health status directly provided by the patient without external interpretation. Thus, instruments assessing GSI meet the core definition of PROMs, since they measure individuals’ self-perceptions and experiences regarding their genitalia—a subjective and person-centered construct. This conceptual framework aligns with the definitions of PROMs established in the literature and underscores the importance of applying PROM-specific methodological frameworks (Mokkink et al., 2016).

### Genital Self-Image, Assessment Measures, and Contributions to the Current Review

This systematic review is based on Berman et al.’s (2003) definition of GSI as a construct that includes feelings, attitudes, thoughts, and behaviors related to the genitals. GSI may influence sexual satisfaction, affect self-esteem, and increase anxiety about sexual performance. In men, GSI plays a significant role in sexual function (Gaither et al., 2017; da Silva et al., 2023). In women, it may influence the pursuit of regular gynecologic care due to concerns about having a healthcare provider visually examining their genitalia (Demaria et al., 2012).

PROMs are commonly used to assess GSI (Arruda et al., 2021; Davis et al., 2013; Herbenick & Reece, 2010; Herbenick et al., 2011; Veale et al., 2013). In addition, this measure plays an important role in research and clinical practice by focusing assessment based on the patients’ self-reported health status of a particular group or population (de Vet et al., 2011). Although PROMs are valuable for assessing GSI, their measurement properties may vary between different contexts of use and populations (Davis et al., 2013; Demaria et al., 2012; Herbenick et al., 2013). Therefore, careful selection of PROMs with appropriate measurement properties and consideration of their clinical applicability are essential steps to ensure that the information collected is valid, reliable, and responsive (Mokkink et al., 2016).

To assess the quality of measurement properties of PROMs in a systematic review, the Consensus-based Standards for the selection of health Measurement INstruments (COSMIN) initiative developed guidelines (Mokkink et al., 2024) to guide the selection and assessment of PROMs according to their studies on measurement properties. Ideally, PROMs should have measurement properties that are sufficient for the construct, population of interest, and context of use (Mokkink et al., 2016).

Conducting a systematic review of PROMs that assess GSI is essential for two reasons. First, it is critical to use high-quality instruments with sufficient measurement properties to assess GSI. This ensures the validity, reliability, and responsiveness of the PROMs for clinical and scientific contexts (Mokkink et al., 2016). Second, although several PROMs are currently used to assess GSI in men and women (Davis et al., 2013; Herbenick & Reece, 2010; Herbenick et al., 2013), these PROMs were developed or evaluated in different populations, such as female university students (Pakpour et al., 2014) and Brazilian men (Arruda et al., 2021), applied on paper (Saffari et al., 2016; Veale et al., 2013; Zielinski et al., 2012) or online (Herbenick et al., 2013), which may raise questions about the selection of the most appropriate PROM.

In addition, systematic reviews are essential to provide a comprehensive and detailed overview of the measurement properties of PROMs and to support evidence-based recommendations for selecting the most appropriate PROM for a construct, population of interest, and context of use (Mokkink et al., 2024). Therefore, the aim of this systematic review is to identify and assess the measurement properties, feasibility, and interpretability of PROMs assessing GSI in women and men to support evidence-based recommendations for the selection of the most appropriate PROM.

## Method

This systematic review followed the PRISMA-COSMIN (Preferred Reporting Items for Systematic Reviews and Meta-Analyses) guidelines for reporting systematic reviews of measurement properties of measurement instruments (Elsman et al., 2024) and the COSMIN methodology for conducting systematic reviews of measurement properties (Mokkink et al., 2024). This methodology consists of eight steps: writing the protocol (Steps 1–3), compiling the search results of the included articles (Step 4), creating a table with an overview of all available PROMs (Step 5), analyzing the evidence for each measurement property by PROM (Step 6), and writing the article (Steps 7 and 8). The protocol has been registered in PROSPERO (registration number CRD42023495616).

### Research Strategy

Keywords and MeSH terms from the literature were collected to develop the search strategy. The following descriptors were combined for each database: “genital self-image,” “genital image,” “genital,” “genital perception,” “genital appearance,” and “genital self-image.” The search filter for measurement properties of measurement instruments developed by COSMIN (Terwee et al., 2012) was applied to the databases. The initial search was conducted in November 2023 and updated in September 2024 in the following databases: Medline (via Ovid), Embase (via Ovid), Web of Science (WoS), SciELO, and CINAHL PLUS (EBSCO interface). Studies published since 1986 were searched. We chose this date because the term “genital identity” was defined for the first time by Waltner (1986).

We also conducted a manual search of the first ten pages of Google Scholar. In addition, we searched articles in PubMed and PROSPERO to identify previously published reviews or papers on the measurement properties of PROMs for GSI. Existing reviews were planned to be excluded and their references analyzed, but no systematic review on the topic was found.

### Eligibility

Eligibility criteria were defined according to the COSMIN guideline for systematic reviews (Mokkink et al., 2024) based on four essential components of the purpose of the review: 1) The PROM must assess the construct of interest, i.e., GSI; 2) the study sample must reflect the population of interest, i.e., women and men aged 16 years and older. The age limit was defined because this is the legal age for providing informed consent to participate in research in some European countries; 3) the focus of the study must be on PROMs; and 4) the aim of the study must involve the assessment of one or more measurement properties or the development of unidimensional PROMs (where the items in a scale or subscale measure a single construct) or of multidimensional PROMs.

We excluded studies available only as abstracts, duplicates, book chapters, clinical trials, and studies that used a PROM but did not intend to evaluate its measurement properties or the PROM was used only as a comparative tool in the validation of another instrument.

All subsequent steps involved two independent reviewers and, when necessary, a third reviewer to resolve discrepancies or a lack of consensus. In addition, studies conducted by the authors of this systematic review were assessed at all stages by a third reviewer not associated with the authorship of the studies. This approach was designed to reduce potential conflicts of interest.

### Screening and Selection of Studies

The databases were searched manually, and studies were selected using Endnote Web. Two independent reviewers initially selected the studies. Titles and abstracts identified in the databases were examined. Studies that met the eligibility criteria were then fully assessed. After inclusion of eligible full-text studies, manual searches of the reference lists of all primary studies included in this systematic review were performed to identify potentially eligible studies. In cases where online access to the study or PROM was not available, we sent emails to the corresponding author requesting additional information.

### Data Extraction

Characterization data were extracted from the studies (PROM name, author and year of publication, country, PROM language, test–retest time, population, and sample size), as well as data on measurement properties defined by the COSMIN taxonomy (Mokkink et al., 2006), including content validity, structural validity, internal consistency, criterion validity, cross-cultural validity/measurement invariance, hypothesis testing for construct validity, test–retest reliability, measurement error, and responsiveness.

### Assessment of Methodological Quality

The methodological quality of each included study was assessed in the first stage. The COSMIN Risk of Bias Checklist (RoB COSMIN) was used to assess the methodological quality of the studies included in the systematic review (Mokkink et al., 2024). The RoB COSMIN has 104 items divided into one box with standards for PROM development and nine boxes for the nine measurement properties. Each item in each study was scored using the COSMIN 5-point rating system: “very good,” “adequate,” “doubtful,” “inadequate,” or “not applicable” (Mokkink et al., 2024). Finally, each measurement property of each PROM was summarized in the study. For the overall rating of the methodological quality of each study, the “worst score counts” method recommended by COSMIN was used, taking the lowest score of each standard for each measurement property (Terwee et al., 2012). This approach is essential because poor methodological aspects cannot be compensated by good aspects (Mokkink et al., 2024).

The methodological quality of the test–retest time for assessing GSI was assessed based on the time interval recommended by COSMIN, which considers a 1- to 2-week interval between test and retest as “very good” (Mokkink et al., 2016). This interval strikes a balance between minimizing recall bias and maintaining respondent stability in the measured construct over time. By consensus of the reviewers, a test–retest time of 15–21 days and between 5 and 6 days was rated as “doubtful,” and intervals longer than 21 days or shorter than 5 days were rated as “inadequate.”

### Assessment of the Quality of Evidence

The second stage was to evaluate the consistency of the results of all the available studies on each measurement property, in order to determine the overall quality of a PROM. If consistent, the results were grouped or pooled and compared with established quality criteria to determine whether the measurement property of the PROM was sufficient (+), insufficient (−), or indeterminate (?). The quality of the evidence was then rated as high, moderate, low, or very low (Mokkink et al., 2024).

The third and fourth stages entailed evaluating and synthesizing the evidence. According to the COSMIN methodology (Mokkink et al., 2024), PROMs can be classified into three types of recommendations. If the evidence indicates that a PROM has adequate content validity (at any level) and at least low-quality evidence for sufficient internal consistency, it should be classified as “strongly recommended for use.” Conversely, when there is high-quality evidence that a measurement property is inadequate, the PROM should be classified as “not recommended for use.” In all other cases when we cannot draw a clear conclusion about the PROM because more high-quality research is needed, the PROM is classified as “no conclusion.” The Grading of Recommendations Assessment, Development, and Evaluation (GRADE) approach modified by COSMIN was used for this purpose. This approach takes into account the following factors: (1) risk of bias, which refers to the methodological quality of the studies; (2) inconsistency, which indicates unexplained discrepancies between study results; (3) imprecision, which is represented by the total sample size of the available studies; and (4) indirectness, which involves evidence from populations other than the one of interest in the review. Concerns about the quality of the evidence were added together to determine the quality of the evidence. The quality of the evidence was then downgraded by one or two levels per factor to moderate, low, or very low evidence if there was a risk of bias, inconsistency (unexplained), imprecision (small sample size), or indirect results.

If the results of the studies included in the review were inconsistent, the overall rating was based on the majority of the results, provided that at least 75% of these results were consistent. When less than 75% of the results were consistent, the pooled result was rated as inconsistent (±). When inconsistencies were identified, the strategy was to avoid summarizing the results and not to rate the evidence. In addition, measurement properties classified as indeterminate in the methodological quality assessment were not rated because there was insufficient evidence to make a conclusive assessment.

## Results

### Bibliographic Research

**Fig. 1:**
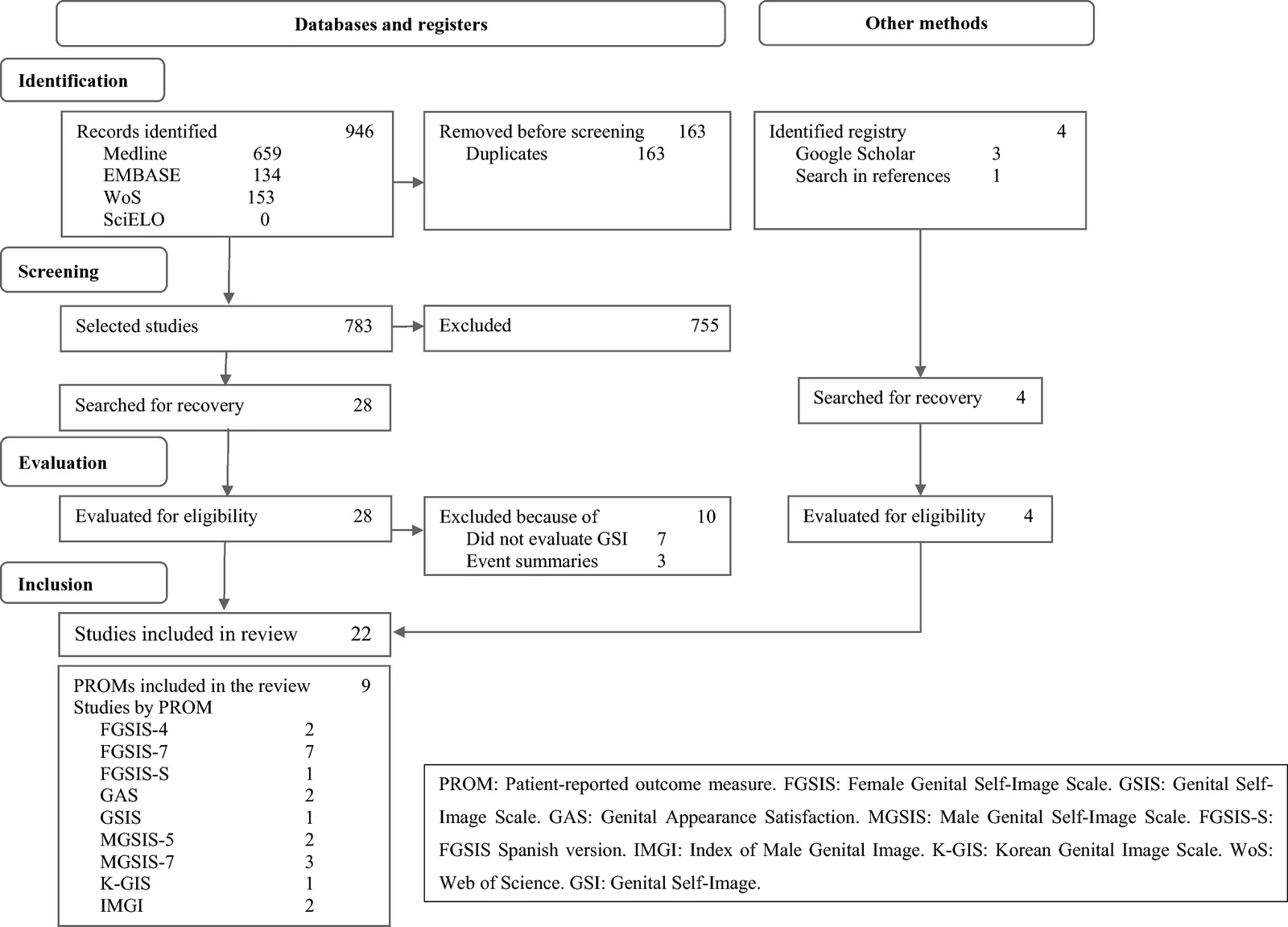
PRISMA-COSMIN 2024 flowchart for systematic reviews of outcome measures

As shown in Fig. 1, a total of 946 studies were identified after searching five databases. After reading the full texts, 18 studies were included. Manual searches of the first 10 pages of Google Scholar and the reference lists of all included studies resulted in four additional studies, for a total of 22 studies and nine PROMs in this systematic review.

### Characteristics of Studies and PROMs

The characteristics of the included studies and PROMs are shown in Table 1. Most studies were conducted in the USA (*n* = 5; 22.73%) and focused on the female population (*n* = 16; 72.73%). Publication years ranged from 2003 to 2023. A total of 14,238 participants were included. All studies reported at least one measurement property.

**Table 1:** Characteristics of the studies and PROMs included (n = 22)

PROM	Author (year)	Country	PROM language	Test–retest time	Population (sample size)
FGSIS (4 items)	Herbenick et al. (2011)	USA	American English	Approximately 2 weeks	Women aged 18–60 (*n* = 1981)
	Mohammed and Hassan (2014)	Egypt	Arabic	2 weeks	Women aged 18–60, married and with a sexually active partner, able to read and understand Arabic or English, with no existing serious medical conditions that would impair daily and occupational function (Content validity: 30 nurses. Other measurement properties: 244)
FGSIS (7 items)	Herbenick et al. (2011)	USA	American English	Approximately 2 weeks	Women aged 18–60 (*n* = 1981)
	Herbenick and Reece (2010)	USA	American English	Approximately 2 weeks	Women attending sex toy parties at home (*n* = 1937)
	DeMaria et al. (2012)	USA	American English	NR	Female university students enrolled in health courses (*n* = 450)
	Ellibes Kaya et al. (2019)	Turkey	Turkish	2 weeks	Health workers, their families, and friends (Content validity: 7 experts and 10 women. Other measurement properties: 461)
	Arruda et al. (2023)	Brazil	Brazilian Portuguese	10 to 14 days	Brazilian women ≥ 18 years (Content validity: 8 experts and 26 women. Other measurement properties: 614)
	Komon et al. (2022)	Thailand	Thai	2 weeks	Sexually active women who were able to communicate fluently in Thai (Structural validity: 336, Reliability: 46)
	Pakpour et al. (2014)	Iran	Persian	2 weeks	Female students ≥ 18 years old, able to read and understand Persian, married and sexually active in the last 6 months (Content validity: 42 female students. Other measurement properties: 1.877)
FGSIS-S (6 items)	Bartolomé et al. (2022)	Spain	Spanish	7–14 days	Native Spanish speakers, ≥ 16 years old and attending gynecological consultations for non-vulvar pathology (Content validity: 5 gynecological consultants, 10 women, 5 sexology specialists, 20 women. Other measurement properties: 202)
GAS	Veale et al. (2013)	UK	British English	NR	Labiaplasty group: Women desiring labiaplasty (*n* = 55). Control group: Women who were undergoing a non-cosmetic gynecologic surgical procedure or in a community setting—not desiring labiaplasty (*n* = 70). Women ≥ 18 years and proficient in English
	Bramwell and Morland (2009)	UK	British English	NR	Women ≥ 16 years (Content validity: 51 women. Other measurement properties: 135)
GSIS	Zielinski et al. (2012)	USA	American English	NR	Study 1: Nulliparous women, ≥ 18 years old, attending an undergraduate course in women's studies. Study 2 and 3: women with prolapse (≥ stage II). Study 4: women who had a vaginal delivery with significant trauma to the genital tract (Content validity: 5 experts. Other measurement properties: 277)
MGSIS (5 items)	Saffari et al. (2016)	Iran	Persian	15 days	Married Iranian men ≥ 18 years (*n* = 1764)
	Herbenick et al. (2013)	USA	American English	Approximately 2 weeks	Men aged 18–60 (*n* = 1047)
MGSIS (7 items)	Herbenick et al. (2013)	USA	American English	Approximately 2 weeks	Men aged 18–60 (*n* = 1047)
	Arruda et al. (2021)	Brazil	Brazilian Portuguese	14–20 days	Brazilian men ≥ 18 years (Content validity: 10 experts and 10 men. Other measurement properties: 518)
	Koçak et al. (2023)	Turkey	Turkish	15 days	Turkish men aged 18–60 (Content validity: 12 experts. Other measurement properties: 336)
IMGI	Davis et al. (2013)	Canada	American English	NR	NR (*n* = 636)
	Omar et al. (2016)	Egypt	Arabic	NR	Egyptian men (*n* = 200)
K-GIS	Lim and Cho (2018)	South Korea	NR	NR	Married women (phase 1, *n* = 168), two physicians, and Korean women (phase 2, *n* = 371)

PROM, Patient-reported outcome measure; FGSIS, Female Genital Self-Image Scale; GSIS, Genital Self-Image Scale; GAS, Genital Appearance Satisfaction; MGSIS, Male Genital Self-Image Scale; FGSIS-S, FGSIS Spanish version; IMGI, Index of Male Genital Image; K-GIS, Korean Genital Image Scale; NR, Not reported

### PROMs Development Studies

Six studies (27.27%) reported on the development of the PROM. In terms of the methodological quality of the overall development requirements, only the GSIS (Berman et al., 2003) was rated “inadequate” for concept elicitation because the PROM development study was not conducted on a representative sample of the target population. In addition, the study did not use an appropriate qualitative data collection method to identify relevant items. All other studies were rated “doubtful” for concept elicitation and overall PROM design evaluation. This rating was based on a lack of clarity in several standards. Among the most recurring issues were doubts about whether the sample was representative of the target population for which the PROM was developed, raising concerns about the generalizability of the results. In addition, there were questions about whether an appropriate approach to data analysis was used, which has a direct impact on the validity of the conclusions drawn. Another critical issue was the uncertainty regarding the independent coding of at least part of the data. It was also not clear whether data collection continued until saturation was reached, which may have limited the full collection of relevant information and affected the depth of the conclusions. Information on the development of the included studies and the assessments for each PROM is presented in Table S1.

### Female Genital Self-Image Scale (FGSIS)

The 7-item version of the FGSIS was developed in the USA (Herbenick & Reece, 2010) and assessed in a second study (Herbenick et al., 2011) for use in American women. For content validity, most studies were rated as having “doubtful” or “inadequate” methodological quality for relevance, comprehensiveness, and comprehensibility, and the results were considered indeterminate. This is due to various reasons. Firstly, patients and professionals were not asked about the relevance, comprehensiveness, and comprehensibility of the PROM, or that it was unclear what approach was used and whether it was appropriate. Only the study by Arruda et al. (2023) had a “very good” rating for methodological quality and sufficient results. In addition, most studies did not examine the comprehensibility of the items by professionals. Structural validity was rated as “doubtful” in only two studies (Herbenick & Reece, 2010; Komon et al., 2022) because they only performed principal component analysis (PCA). Only Ellibes Kaya et al. (2019) and Herbenick et al. (2011) were classified as having insufficient results, as the confirmatory factor analysis (CFA) showed Comparative Fit Index (CFI) values lower than 0.95 and Root Mean Square Error of Approximation (RMSEA) values higher than 0.06. All other studies had results considered sufficient. For internal consistency, the methodological quality was classified as “doubtful” (Ellibes Kaya et al., 2019; Jansuwan et al., 2022; Komon et al., 2022; Loewinski et al., 2022) due to a lack of evidence for the unidimensionality of the PROM or each subscale; the results were classified as indeterminate because the criterion “at least low evidence for sufficient structural validity” was not met (Jansuwan et al., 2022; Komon et al., 2022; Loewinski et al., 2022). For Ellibes Kaya et al. (2019) and other studies (Arruda et al., 2023; Demaria et al., 2012; Herbenick & Reece, 2010; Herbenick et al., 2011; Pakpour et al., 2014), the results were considered sufficient. Only one study (Arruda et al., 2023) received a “very good” rating for methodological quality for test–retest reliability, while others were rated “doubtful” because it was unclear whether the patients were stable, whether the conditions were similar between test–retest, or whether the correlation coefficient was calculated without evidence that no systematic changes had occurred. All studies had sufficient results for test–retest reliability. For measurement error, the methodological quality was “doubtful” (Ellibes Kaya et al., 2019), as there is no defined minimally important change (MIC) in the literature to assess the measurement error of GSI. Only Arruda et al. (2023) received a “very good” rating; consequently, the results were rated as indeterminate.

For the results of construct validity by instrument comparison (Arruda et al., 2023; Demaria et al., 2012; Ellibes Kaya et al., 2019; Herbenick & Reece, 2010; Jansuwan et al., 2022; Komon et al., 2022; Pakpour et al., 2014), the quality rating was “very good” and the results were classified as sufficient. However, assessments by known groups (Demaria et al., 2012; Ellibes Kaya et al., 2019; Herbenick & Reece, 2010) had “doubtful” methodological quality, primarily due to a lack of description of important subgroup characteristics. Nevertheless, the results were classified as sufficient because they aligned with the hypothesis. The overall rating and quality of evidence for the FGSIS-7 were sufficient and had high quality for content validity, internal consistency, construct validity (comparison between instruments), and test–retest reliability. However, structural validity was inconsistent, as 75% of the studies did not have “very good” or “adequate” methodological quality. Measurement error was classified as indeterminate because only one study with “very good” methodological quality assessed this measure property and was classified as indeterminate. Consequently, the overall rating was indeterminate.

The 4-item version was developed and its measurement properties were assessed in the same study by Herbenick et al. (2011) and validated for Egypt by Mohammed and Hassan (2014). Content validity was not assessed (Herbenick et al., 2011), nor was structural validity (Mohammed & Hassan, 2014), measurement invariance, or measurement error (Herbenick et al., 2011; Mohammed & Hassan, 2014). The overall classification and quality of evidence for the FGSIS-4 were sufficient and had high quality for structural validity, internal consistency, and construct validity (comparison between instruments). It was not possible to determine the overall rating and quality of evidence for content validity, construct validity (comparison between known groups), and test–retest reliability because there were no studies with “very good” or “adequate” methodological quality.

The FGSIS Spanish version (FGSIS-S) (Bartolomé et al., 2022) has 6 items. The authors did not assess measurement invariance and measurement error. For content validity, the methodological quality was rated as “doubtful” or “inadequate” for relevance and comprehensiveness, as these aspects were not assessed by patients. Patient comprehension and professional ratings of relevance, comprehensiveness and comprehensibility were also rated as “doubtful.” This was due to a lack of clarity about whether appropriate methods were used, whether an interview guide was used, and whether professionals from all relevant disciplines were involved; consequently, the results were classified as indeterminate because only one study evaluated this measurement property, and it was deemed “inadequate” in terms of methodological quality. Structural validity was “doubtful” and sufficient, as only PCA was performed. Internal consistency was assessed as “very good” and considered sufficient. Test–retest reliability was “doubtful” because it was unclear whether patients were stable and the correlation coefficient was calculated without evidence that no systematic change had occurred, but sufficient because the intraclass correlation coefficient or weighted Kappa ≥ 0.70. The hypothesis test by comparison between known groups was classified as “doubtful” because an adequate description of the important characteristics of the subgroups analyzed was not provided, but “sufficient” because the result was in agreement with the hypothesis. The overall classification of the FGSIS-S was considered sufficient for structural validity, internal consistency, construct validity (comparison between known groups), and test–retest reliability. However, the quality of evidence was rated as high only for internal consistency. For the other measurement properties, the quality of the evidence was rated as low due to a serious risk of bias, as the study was deemed methodologically “doubtful.”

### Male Genital Self-Image Scale (MGSIS)

The 7-item version of the MGSIS was developed in the USA (Herbenick et al., 2013) to assess male GSI, but content validity, test–retest reliability, measurement error, and hypothesis testing for construct validity were not assessed. The methodological quality of structural validity was assessed as “doubtful” because it was unclear whether the sample size included in the analysis was adequate. However, the result was classified as sufficient because the scale was unidimensional. Internal consistency (*α* = 0.93) was classified as “very good” and sufficient since it met all the criteria. In the Brazilian (Arruda et al., 2021) and Turkish studies (Koçak et al., 2023), content validity, structural validity, internal consistency, test–retest reliability, and hypothesis testing for construct validity were evaluated. For the methodological quality, content validity for relevance, comprehensiveness, and comprehensibility as assessed by patients was “inadequate” (Koçak et al., 2023) and “doubtful” (Arruda et al., 2021), while relevance and comprehensiveness as assessed by professionals were “inadequate” (Koçak et al., 2023) and “doubtful” (Arruda et al., 2021), respectively. The results were indeterminate (Koçak et al., 2023) due to insufficient information reported; however, they were sufficient for the Brazilian study (Arruda et al., 2021). Structural validity and internal consistency were “very good,” with a rating of sufficient. Test–retest reliability was “doubtful,” as the test–retest time exceeded 14 days, but the results were sufficient. Consequently, measurement error was rated “doubtful” in the Brazilian study and was indeterminate due to the absence of a MIC. The comparison of instruments for construct validity was “very good” with sufficient results. The overall classification and quality of the evidence for the MGSIS-7 were sufficient and high for structural validity, internal consistency, and construct validity (comparison between known groups). Content validity, test–retest reliability, and measurement error could not be determined due to the lack of studies with “very good” or “adequate” methodological quality.

For the 5-item version, patients assessed the methodological quality of content validity as “doubtful” for comprehensibility and “inadequate” for relevance and comprehensiveness. For professionals, the quality was “inadequate” for relevance, comprehensiveness, and comprehensibility. This is because the method used to question professionals and patients was also “inadequate.” It was not explicitly asked whether the items together fully capture the construct the PROM is intended to measure, or whether the domains included together capture the broader construct measured by the PROM’s total score. The results were indeterminate (Saffari et al., 2016). Structural validity was “doubtful” due to unclear sample size (Herbenick et al., 2013) and “very good” (Saffari et al., 2016), with both studies rated as sufficient. Internal consistency was “doubtful” for Herbenick et al. (2013) and sufficient for both studies. Only Saffari et al. (2016) assessed measurement invariance. The study had “very good” methodological quality, and the results were sufficient. Test–retest reliability was “doubtful” for both studies because the test–retest time exceeded 14 days (Saffari et al., 2016) and it was unclear whether patients were stable (Herbenick et al., 2013). The classification for test–retest reliability was insufficient only for Herbenick et al. (2013) because the correlation was < 0.70. Construct validity by instrument comparison was deemed “inadequate” due to the inappropriate statistical method, which was not reported. However, the results were classified as sufficient as they aligned with the hypotheses (Herbenick et al., 2013). In the study by Saffari et al. (2016), both comparison by instruments and by known groups was “very good” and sufficient. For structural validity, internal consistency, measurement invariance, and construct validity (comparison between instruments and known groups), the overall classification and quality of evidence for the MGSIS-5 were sufficient and high, respectively. For content validity and test–retest reliability, there were only studies with “doubtful” methodological quality; therefore, it was not possible to determine the overall rating and quality of evidence.

### Genital Self-Image Scale (GSIS)

The GSIS was developed by Berman et al. (2003) to assess female GSI, but measurement properties were not assessed. Zielinski et al. (2012) conducted validation with women in the USA, but did not evaluate the measurement invariance. The methodological quality of content validity was “inadequate” for relevance, comprehensiveness, and comprehensibility by patients, primarily because the qualitative method used was not considered adequate to comprehensively assess the construct; it was “doubtful” for relevance and comprehensiveness by professionals because it was unclear whether at least two researchers were involved in the analysis; consequently, the results were classified as indeterminate because only one study evaluated this measurement property. The structural validity was considered “doubtful” because only one PCA was performed. However, the results were sufficient. The internal consistency and test–retest reliability were also considered “doubtful” because the dimensionality of the scale was not assessed. However, the results were sufficient. Measurement error was “doubtful” because it was unclear whether the patients were stable, whether the time interval was appropriate, and whether the conditions in the test and retest were similar. The result for measurement error was considered indeterminate because there is no defined MIC. Construct validity was “doubtful” and sufficient because it did not adequately describe the important characteristics of the subgroups analyzed, but the results were in accordance with the hypotheses. The overall ratings for content validity and measurement error were considered indeterminate because only one study was available, assuming that the data were consistent. Therefore, the quality of evidence could not be rated according to the GRADE system. For structural validity, internal consistency, construct validity (comparison between instruments), and test–retest reliability, the overall rating was sufficient but of low quality due to a serious risk of bias, as the study was methodologically classified as “doubtful.”

### Genital Appearance Satisfaction (GAS)

This PROM was developed by Bramwell and Morland (2009) to assess GSI in women, and it was validated for the UK (Veale et al., 2013). None of the studies assessed the measurement invariance, test–retest reliability, or measurement error of the GAS. For content validity, the methodological quality was “doubtful” for patient relevance and comprehensibility, as the method was doubtful and the number of patients was < 30; it was assessed “inadequate” for patient and professional relevance and comprehensiveness, as the method used was also considered inadequate. The results for content validity were considered indeterminate. The structural validity for Veale et al. (2013) was “inadequate” and the results were insufficient, as the sample was less than five times the number of items. For Bramwell and Morland (2009), the quality and results were “doubtful” and “indeterminate,” respectively, because only PCA was conducted and insufficient information was reported. The internal consistency for Veale et al. (2013) was “very good,” but was insufficient due to the lack of low-quality evidence for sufficient unidimensionality. For Bramwell and Morland (2009), internal consistency was “very good” and sufficient. Construct validity by comparison of instruments was “adequate” for Veale et al. (2013), because although the measurement properties of the comparison instruments were sufficient, there were uncertainties about their applicability to the study population, but it was sufficient. The comparison between known groups was “very good” and sufficient (Veale et al., 2013). For Bramwell and Morland (2009), the construct validity of the comparator instruments was “very good” and sufficient. The overall rating for internal consistency of the GAS was inconsistent, as 75% of the studies did not have “very good” or “adequate” methodological quality, but the quality of evidence was rated as high. For construct validity (comparison between instruments and known groups), the overall classification was sufficient with high quality. For content and structural validity, it was not possible to determine the classification, as the available studies were of “doubtful” or “inadequate” methodological quality.

### Index of Male Genital Image (IMGI)

The IMGI was originally developed in Canada (Davis et al., 2013) for use by the male population and was later validated in Egypt (Omar et al., 2016). No study assessed the content validity, measurement invariance, test–retest reliability, or measurement error of the IMGI. For the Canadian male population (Davis et al., 2013), the methodological quality of structural validity was “doubtful” and the results were insufficient because only a PCA was performed. Structural validity was assessed as “doubtful” but sufficient for the Egyptian study (Omar et al., 2016). The internal consistency was assessed as “doubtful,” and the results were deemed indeterminate because dimensionality was not evaluated (Davis et al., 2013). In the Egyptian study, the internal consistency was assessed as “very good;” however, the results were deemed insufficient because one factor had a Cronbach’s alpha below 0.70 (Omar et al., 2016). Construct validity through instrument comparison was “inadequate” for Davis et al. (2013), as no information was provided on the measurement properties of the comparator instruments, and “very good” for Omar et al. (2016). Both studies were considered sufficient for this measurement property. Davis et al. (2013) also conducted a comparison of known groups, which was “doubtful” because the important characteristics of the subgroups were not adequately described and the statistical methods used were not ideal, but the results were sufficient to agree with the hypotheses. The overall rating for internal consistency was insufficient, but the quality of the evidence was high for internal consistency and construct validity (comparison between instruments). It was not possible to determine the overall rating and quality of evidence for structural validity and construct validity (comparison between known groups) due to the lack of studies with “very good” or “adequate” methodological quality.

### Korean Genital Image Scale (K-GIS)

The K-GIS (Lim & Cho, 2018) was developed for women in South Korea. However, content validity, measurement invariance, test–retest reliability, and measurement error were not assessed. Structural validity and construct validity (comparison between instruments) were “very good” and sufficient. Internal consistency was “inadequate” because dimensionality was not assessed, but it was rated as sufficient due to the presence of at least low-quality evidence of sufficient structural validity and adequate internal consistency coefficients. The overall rating of the K-GIS was sufficient for structural validity, internal consistency, and construct validity. However, the quality of evidence for internal consistency was rated as low due to the risk of bias.

### Interpretability and Viability of PROMs

The interpretability of the PROMs was limited because of a lack of data in the studies. The authors did not report data on floor and ceiling effects, benchmark scores, credibility scores, or information on the MIC for GSI. Only one study for the FGSIS-7 (Arruda et al., 2023) and one for the MGSIS-7 (Arruda et al., 2021) reported cutoff scores, but these data were insufficient to conduct a robust analysis of the discriminative ability of the PROMs.

Feasibility was assessed for all PROMs (Table 2) and was high, as most are relatively short, available in multiple languages, and flexible for online or paper administration. Most instruments do not report the time required for completion, with the exception of the FGSIS-7, which takes approximately 2 min, and the IMGI, which takes approximately 10 min. The number of items varies from 4 to 37, with shorter instruments being particularly feasible in studies with multiple measures or large samples. Full copies of all PROMs are available and free to access and license. Only the FGSIS-7 and MGSIS-7 have been translated into three or more languages.

**Table 2:** PROMs viability

Name (version)	Burden on patients		Practical aspects for obtaining PROM				
	Filling time	Number of items	Full copy available	Licensing information	Costs	Form of administration	Translations
FGSIS (4 items)	NR	4	Yes	Free	Free	Online and paper	English and Arabic
FGSIS (7 items)	Less than 2 min	7	Yes	Free	Free	Online and paper	English, Turkish, Brazilian Portuguese, Thai, and Persian
FGSIS-S (6 items)	NR	6	Yes	Free	Free	Online	Spanish
GAS	NR	11	Yes	Free	Free	Paper	English
GSIS	NR	20	Yes	Free	Free	Paper	English
MGSIS (5 items)	NR	5	Yes	Free	Free	Paper	English and Persian
MGSIS (7 items)	NR	7	Yes	Free	Free	Online	English, Brazilian Portuguese, and Turkish
IMGI	Less than 10 min	14	Yes	Free	Free	Paper	English and Arabic
K-GIS	NR	37	Yes	Free	Free	Paper	NR

FGSIS, Female Genital Self-Image Scale; GSIS, Genital Self-Image Scale; GAS, Genital Appearance Satisfaction; MGSIS, Male Genital Self-Image Scale; FGSIS-S, FGSIS Spanish version; IMGI, Index of Male Genital Image; K-GIS, Korean Genital Image Scale; NR, Not reported

### Data Synthesis

Information on the methodological quality of the included studies and the ratings of each measurement property for each PROM is presented in Tables S1–S9. The overall classification of measurement properties and quality of evidence for the nine PROMs are presented in Table 3. No PROM had all of the measurement properties assessed, and there was insufficient evidence to draw clear and definitive conclusions for recommending or not recommending a PROM. For the assessment of female GSI, only for the FGSIS-7 the quality of the evidence for content validity could be assessed and its use could be considered potentially recommended. For the male population, no PROM had at least low-quality evidence for content validity to be potentially recommended according to COSMIN.

**Table 3:** Evidence synthesis for PROMs on GSI

Measure properties	FGSIS	FGSIS	FGSIS-S	GAS	GSIS	MGSIS	MGSIS	IMGI	K-GIS
	(4 items)	(7 items)	(6 items)			(5 items)	(7 items)		
Content validity									
Number of studies	1	5	1	1	1	1	2		
Rating	NE	+	?	NE	?	NE	NE		
QoE	NE	High	NE	NE	NE	NE	NE		
Structural validity									
Number of studies	1	7	1	2	1	2	3	2	1
Rating	+	±	+	NE	+	+	+	NE	+
QoE	High	High	Low	NE	Low	High	High	NE	High
Internal consistency									
Number of studies	1	9	1	2	1	2	3	2	1
Rating	+	+	+	±	+	+	+	-	+
QoE	High	High	High	High	Low	High	High	High	Low
Validity/cross-cultural invariance of the measure									
Number of studies						1			
Rating						+			
QoE						High			
Hypothesis testing for construct validity – comparison of instruments									
Number of studies	2	7		2		2	1	2	1
Rating	+	+		+		+	+	+	+
QoE	High	High		High		High	High	High	High
Hypothesis testing for construct validity – comparison of known groups									
Number of studies	1	3	1	1	1	1		1	
Rating	NE	NE	+	+	+	+		NE	
QoE	NE	NE	Low	High	Low	High		NE	
Test–retest reliability									
Number of studies	2	5	1		1	2	2		
Rating	NE	+	+		+	NE	NE		
QoE	NE	High	Low		Low	NE	NE		
Measurement error									
Number of studies		2			1		1		
Rating		?			?		NE		
QoE		NE			NE		NE		

GSI, Genital Self-Image; FGSIS, Female Genital Self-Image Scale; GSIS, Genital Self-Image Scale; GAS, Genital Appearance Satisfaction; MGSIS, Male Genital Self-Image Scale; FGSIS-S, FGSIS Spanish version; IMGI, Index of Male Genital Image; K-GIS, Korean Genital Image Scale; QoE, Quality of evidence; NE, not evaluated;?, indeterminate results; + , sufficient results; ± , inconsistent results; −, insufficient results

For the assessment of female GSI, only the FGSIS-7 showed potential for recommendation, as it provided sufficient evidence of content validity.

## Discussion

This systematic review included 22 articles, and nine PROMs assessing GSI were assessed for risk of bias, measurement properties, and quality of evidence according to the COSMIN methodology (Mokkink et al., 2024). No study evaluated all the measurement properties of a PROM, and none stood out in terms of quality of evidence. Therefore, it is difficult to recommend a PROM for the assessment of GSI, as no definitive conclusions could be drawn from the PROMs. However, the FGSIS-7 showed potential for recommendation. Although it is a promising PROM, further research is needed to assess its measurement properties. It is recommended that its structural validity, cross-cultural validity/measurement invariance, and measurement error are investigated.

In addition to its relevance for instrument validation and selection, using PROMs to assess GSI has direct clinical and public health implications. These instruments can support the evaluation of patient perceptions before and after genital-related surgeries, such as genital reconstruction procedures, gender affirmation surgeries, and vaginoplasty (Alavi-Arjas et al., 2023; Kloer et al., 2023; Minikowski et al., 2025). PROMs can also inform sexual and reproductive health promotion strategies, providing clinicians with patient-centered metrics to address concerns about genital appearance and function. Furthermore, PROMs can help identify the psychosocial impact of conditions such as erectile dysfunction and dyspareunia, guiding tailored interventions. According to Prinsen et al. (2016), validated and reliable instruments are essential to ensure robust and reliable results. PROMs play a critical role in clinical practice and research because they can determine how much GSI is positive for patients (Han et al., 2023). In this systematic review, the focus was exclusively on PROMs to assess GSI, following the COSMIN methodology to provide an overall view of these instruments and their measurement properties. Among the nine PROMs, many important measurement properties were either missing or inadequately assessed. Researchers can decide if it is necessary to develop more PROMs to evaluate GSI and conduct detailed evaluations of their measurement properties, especially for the FGSIS-7. This will provide both quantitative evidence of quality and qualitative insights through content validity (relevance, comprehensiveness, and comprehensibility by professionals and patients) to allow better comparability between PROMs. In addition, the interpretability of PROMs should also be assessed, as this will allow clinical meaning to be assigned to the quantitative scores or changes in scores of the instruments.

Among the included PROMs in this systematic review, only the MGSIS-7 (Arruda et al., 2021; Herbenick et al., 2013; Koçak et al., 2023) and the FGSIS-7 (Arruda et al., 2023; Demaria et al., 2012; Ellibes Kaya et al., 2019; Herbenick & Reece, 2010; Herbenick et al., 2011; Komon et al., 2022; Pakpour et al., 2014) were translated and assessed in more than three languages. Future studies are needed to assess the cross-cultural validity/measurement invariance of these PROMs in other contexts (e.g., community people, students, etc.). The limited availability of translations can be attributed to several factors. First, concern about GSI is relatively new, with the term first introduced in the literature in 1986 (Waltner, 1986), and most of the articles included in this systematic review were published between 2009 and 2023, with a focus on the last 7 years. This suggests that the development of PROMs for GSI is still in its early stages. Second, the available PROMs are limited in their assessment of measurement properties, which makes it difficult to assess their quality and conduct translation research in other contexts. Of the 22 included studies, only eight evaluated more than five measurement properties (Arruda et al., 2021, 2023; Bartolomé et al., 2022; Ellibes Kaya et al., 2019; Komon et al., 2022; Pakpour et al., 2014; Saffari et al., 2016; Zielinski et al., 2012). However, the PROMs included in this systematic review are brief and have standardized scoring rules, which facilitate their use, as lengthy and complex PROMs may discourage their use and affect their cross-cultural applicability (Han et al., 2023).

According to COSMIN, for a PROM to be potentially recommended, it must have at least very low-quality evidence of sufficient content validity (Mokkink et al., 2024). Content validity is the most important measurement property of a PROM, as the lack of high-quality evidence in this area may compromise other measurement properties (Terwee et al., 2018). This measurement property is assessed by asking patients and professionals from different disciplines about the relevance, comprehensiveness, and comprehensibility of the items, instructions, response options, and recall period (Mokkink et al., 2016). In this systematic review, only the FGSIS-7 had high-quality evidence and sufficient results to support a recommendation. However, most of the studies were “doubtful” or had “inadequate” methodological quality. The low methodological quality of the studies and the classification of the results as uncertain in studies reporting on the development of PROMs made it impossible to determine the quality of evidence for most PROMs. According to COSMIN, it is essential that the development process of a PROM and the validity of its content are reported in detail (Mokkink et al., 2024). Future studies using cognitive interviews should clearly specify whether all aspects of the PROM (e.g., instructions, items, response options, and recall period) were evaluated with an adequate sample size of participants and professionals (Prinsen et al., 2018).

Structural validity and internal consistency are closely related because each factor identified by structural validity must be individually assessed for internal consistency using appropriate statistical methods (Mokkink et al., 2024). In PROMs based on reflective models, it is essential to assess these measurement properties in order to determine the number of factors and the items corresponding to each factor. COSMIN recommends the use of CFA to assess structural validity and Cronbach’s alpha for internal consistency (Mokkink et al., 2024). The lack of CFA and the use of inappropriate statistical values in factor analysis resulted in “doubtful” or “inadequate” methodological quality for the structural validity of most PROMs. This resulted in low-quality evidence for the FGSIS-S and GSIS and a lack of scoring for the GAS and IMGI. Despite these limitations, most PROMs showed high-quality evidence of internal consistency, as Cronbach’s alpha was reported for each factor identified in the factor analysis. In addition, internal consistency was assessed for all PROMs. For the GSIS, the quality of the evidence was considered low due to the “doubtful” methodological quality of the included study. The lack of adequate assessment of structural validity compromised the quality of evidence for internal consistency, as structural validity is an essential prerequisite for this analysis (Mokkink et al., 2024). For each factor, adequate statistics, such as Cronbach’s alpha, are required for a proper assessment of internal consistency (de Vet et al., 2011).

The assessment of cross-cultural validity/measurement invariance was evaluated in only one study for the MGSIS-5 (Saffari et al., 2016), which was sufficient with high-quality evidence. According to COSMIN, this evaluation requires data from two different populations, with one population completing the original version of the instrument and the other completing the culturally adapted version. The lack of cross-cultural validity testing in most of the PROMs included in this systematic review highlights a critical gap in the translation and validation process. The cross-cultural validity is essential to ensure that the translated version measures the same construct as the original version (Mokkink et al., 2024). Therefore, it is highly recommended that translated PROMs undergo cross-cultural validity/invariance testing.

Hypothesis testing for construct validity is useful when comparing the relationship between instruments measuring similar or different constructs or when comparing subgroups of patients (Mokkink et al., 2016). In the case of hypothesis testing involving subgroups, the methodological quality of most studies was “doubtful” due to incomplete information on the characteristics of the groups. This limitation prevented the assessment of the quality of evidence for the FGSIS-4, FGSIS-7, and IMGI. Only the FGSIS-S and GSIS received a sufficient overall rating, but with low-quality evidence due to the risk of bias caused by the “doubtful” methodological quality. However, when comparing different instruments, all PROMs showed high-quality evidence for testing the hypothesis of construct validity, with an overall satisfactory rating. According to Mokkink et al. (2024), hypothesizing in advance is essential for clinicians to predict the expected relationship between instruments or the differences between patient groups regarding the construct measured by the PROM.

In this systematic review, test–retest reliability was assessed for three PROMs, with high-quality evidence found only for the FGSIS-7. Low-quality evidence was found for the FGSIS-S and GSIS. The lack of sufficient information on patient stability led to “doubtful” methodological quality, which increased the risk of bias. According to COSMIN, test–retest reliability should be assessed using repeated measures in participants who remain stable between assessments (Mokkink et al., 2016, 2024).

No PROM was assessed for the quality of evidence regarding measurement error. This measurement property is assessed on the basis of two measurements taken on the same subject at different times. For this assessment, each study must clearly present the calculation and value of the MIC, the standard error of measurement, the smallest detectable change (SDC), and the limits of agreement (LoA). According to COSMIN, the MIC is defined as “the smallest change in the construct score that patients perceive as important” and can be determined from different studies (Mokkink et al., 2024). To receive a sufficient general classification and high-quality evidence regarding measurement error, the SDC or LoA must be smaller than the MIC. In addition, the risk of bias, inconsistency, imprecision and indirectness, as assessed by the modified GRADE, must be satisfactory (Mokkink et al., 2024). However, only the FGSIS-7, GSIS, and MGSIS-7 assessed measurement error, but received an indeterminate overall rating and the quality of evidence was not defined because these requirements were not met, as there is no established MIC for GSI in the literature.

Criterion validity and responsiveness were the only measurement properties not assessed for any of the PROMs in this systematic review. There is no established gold standard for PROMs assessing GSI. However, according to COSMIN (Mokkink et al., 2024), in the absence of a gold standard in the literature, criterion validity can be examined by comparing the long version of a PROM with its short version. Therefore, for MGSIS-7 and FGSIS-7, comparison with their short versions, MGSIS-5 and FGSIS-4, respectively, may be useful for assessing criterion validity in future studies. On the other hand, responsiveness is an essential measurement property for clinical practice because it measures the ability of a PROM to detect changes in the construct over time. This property assists healthcare professionals in monitoring the improvement or deterioration of a patient’s condition following an intervention (Mokkink et al., 2024).

### The Clinical Applicability of PROMs for Genital Self-Image Assessment

PROMs for GSI have relevant and diverse potential clinical applications. One main use is during case conceptualization, particularly in cases of body dysmorphia (Sharp, 2021), elective genital surgery (Applebaum & Placik, 2022; Schmidt & Rowen, 2021), and chronic genital conditions, such as vulvar dermatoses (Rivera et al., 2022). In these cases, negative GSI can be an important indicator of subjective suffering that is often overlooked by physical examinations and traditional biomedical assessments. Studies indicate that genital dissatisfaction is associated with sexual dysfunction and reduced quality of life, suggesting that PROMs for GSI could be valuable complementary tools in clinical evaluations (Rivera et al., 2022; Sharp, 2021).

Additionally, these PROMs can assist in selecting and adapting treatment. Low scores on these scales may indicate the need for specific interventions, such as psychological support, education about genital anatomy, or multidisciplinary follow-up care. Applebaum and Placik (2022), for example, highlight that dissatisfaction with genital appearance is often linked to cognitive distortions and a lack of understanding about anatomical diversity. Measuring these aspects can help screen candidates for genital cosmetic surgery and prevent unnecessary procedures. Another relevant aspect is the use of these PROMs for monitoring processes and evaluating therapeutic outcomes. A systematic review by Alavi-Arjas et al. (2023) revealed that GSI scores significantly improved following surgical and psychological interventions, demonstrating the scales’ sensitivity to clinical changes. These results highlight improvements in genital satisfaction levels following cosmetic and reconstructive procedures, which reinforce the potential of these scales as instruments for evaluating clinical outcomes. Thus, the GSI can be considered not only an outcome, but also a marker of the therapeutic process. Despite the growing interest in using PROMs clinically to assess GSI, there is still a lack of evidence regarding their validity and applicability to trans populations. Since existing scales were developed and validated with cisgender individuals, their ability to capture the specific experiences of transgender individuals, especially in the context of genital dysphoria and gender-affirming interventions, is questionable. As Sharp (2021) discussed, it is necessary to adapt or develop instruments that consider the bodily, social, and subjective nuances of transgender individuals to ensure that such measures adequately reflect their GSI and can act as useful PROMs in this clinical context. These measures should also be systematically included in clinical studies to assess variations in GSI in significant clinical outcomes. Such investigations may establish these tools as useful PROMs, promoting more effective integration of subjective assessments into therapeutic practices.

### Limitations

To the best of our knowledge, this is the first systematic review to comprehensively assess the quality of the measurement properties of PROMs used to evaluate GSI in both men and women. This addresses a significant gap in the literature. However, this systematic review has several limitations. First, the inclusion of studies with populations older than 16 years may limit the generalizability of the findings to younger age groups, as there may be PROMs specifically designed for these populations that were excluded. These instruments may address unique aspects of development and health perceptions that differ significantly from those assessed in older groups, creating important gaps in the application of results to children and adolescents. Second, there were no data to assess the interpretability of PROMs, such as reference scores, ceiling and floor effects, and MIC. According to COSMIN, interpretability is crucial to ensure that the scores obtained are understandable and clinically useful, allowing health professionals and researchers to interpret the results appropriately. The main strengths of this systematic review include the ability to conduct a more rigorous assessment of the methodological quality of the studies, to reduce bias in the analysis of the measurement properties, and to provide a strong basis for recommending the use of PROMs in research and clinical practice. There was no language restriction, which allowed for a more comprehensive and inclusive analysis of tools available in different languages. In addition, several databases and gray literature were consulted using specific search terms and without language restrictions, ensuring a broad inclusion of studies. This systematic review not only synthesizes available evidence but also highlights major methodological gaps, guiding future instrument development and informing clinicians’ choices when selecting PROMs for evaluating GSI.

## Conclusion

This systematic review identified and assessed the methodological quality of nine PROMs for the assessment of GSI. It was not possible to draw definitive conclusions about the PROMs evaluated. For the assessment of female GSI, only the FGSIS-7 showed potential for recommendation, as it provided sufficient evidence of content validity. None of the PROMs for the male population provided even low-quality evidence of content validity, so no recommendations were made. Although the FGSIS-7 appears promising, future studies should collect additional data to evaluate its measurement properties using a robust methodology consistent with established guidelines for conducting PROM measurement properties studies.

## Supplementary Information

Below is the link to the electronic supplementary material.

Supplementary file1 (DOCX 56 KB)

## Data Availability

Data will be made available upon reasonable request.
