# Correction: Patient-Reported Outcome Measures Assessing Genital Self-Image: A Systematic Review of Measurement Properties

**DOI:** 10.1007/s10508-026-03639-3

**Published:** 2026-08-19

**Authors:** Erisvan Vieira da Silva, Guilherme Tavares de Arruda, Leticia Amaral Corrêa, Melissa Medeiros Braz, Hedioneia Maria Foletto Pivetta

**Affiliations:** 1 Postgraduate Program in Movement Sciences and Rehabilitation, Federal University of Santa Maria, Santa Maria, Brazil; 2 Postgraduate Program in Physiotherapy, Federal University of São Carlos, São Carlos, Brazil; 3 Department of Chiropractic, Faculty of Medicine, Health and Human Sciences, Macquarie University, Sydney, Australia; 4 Department of Physiotherapy and Rehabilitation, Federal University of Santa Maria, Avenida Roraima, 1000, Camobi, Santa Maria, RS 97105-900 Brazil

**Correction to: Archives of Sexual Behavior (2026) 55:2009–2025** https://doi.org/10.1007/s10508-026-03488-0

The name of coauthor Leticia Amaral Corrêa was presented as Leticia Correa do Amaral in the article as originally published.

The original article has been corrected.

