# Supplementary material for: Patient-Reported Outcome Measures Assessing Genital Self-Image: A Systematic Review of Measurement Properties
Source: Arch Sex Behav. 2026 Jul 13;55(5):2009–25. doi: 10.1007/s10508-026-03488-0 (PMC13427953; doi:10.1007/s10508-026-03488-0)
Supplement: Supplementary file 1 — Supplementary file1 (DOCX 56 KB) [file 10508_2026_3488_MOESM1_ESM.docx]

**Table S1**

*Quality of PROMs development*

|  | | | **PROM design** | | | | | | **Pilot test** |
| --- | --- | --- | --- | --- | --- | --- | --- | --- | --- |
|  |  |  | **General design requirements** | | | | **Concept elicitation** | **Total PROM design** |  |
| **PROM** | **Reference** | **Language in which PROM was developed** | **Clear construct** | **Clear origin of construct** | **Clear target population for which the PROM was developed** | **Clear context of use** |  |  |  |
| GAS | Bramwell and Morland (2009) | English | Yes | No | Yes | No | D | D | D |
| FGSIS | Herbenick and Reece (2010) | English | Yes | Yes | Yes | No | D | D | NR |
| IMGI | Davis et al., (2013) | English | Yes | No | Yes | No | D | D | NR |
| MGSIS | Herbenick et al., (2013) | English | Yes | Yes | Yes | No | D | D | NR |
| K-GIS | Lim and Cho (2018) | NR | No | Yes | Yes | No | D | D | NR |
| GSIS | Berman et al., (2003) | NR | Yes | Yes | No | No | I | I | NR |

PROM: Patient-Reported Outcome Measure. GAS: Genital Appearance Satisfaction. FGSIS: Female Genital Self-Image Scale. IMGI: Index of Male Genital Image. MGSIS: Male Genital Self-Image Scale. K-GIS: Korean Genital Image Scale. GSIS: Genital Self-Image Scale. NR: Not reported. VG: very good. A: adequate. D: doubtful. I: inadequate.

**Table S2**

*PROMs content validity*

| **PROM** | **Author (year)** | **Ask patients** | | | **Ask professionals** | | | **Rating** | **Rating PROM** |
| --- | --- | --- | --- | --- | --- | --- | --- | --- | --- |
|  |  | **Relevance** | **Comprehensiveness** | **Comprehensibility** | **Relevance** | **Comprehensiveness** | **Comprehensibility** |  |  |
| FGSIS  (7 items) | Loewinski et al., (2022) | I | I | D | D | D | NR | ? | + |
|  | Pakpour et al., (2014) | I | I | I | I | I | NR | ? |  |
|  | Ellibes Kaya et al., (2019) | D | I | D | D | D | D | ? |  |
|  | Arruda et al., (2023) | VG | VG | VG | VG | VG | NR | + |  |
|  | Komon et al., (2022) | D | I | D | D | D | D | ? |  |
| FGSIS-S  (6 items) | Bartolomé et al., (2022) | I | I | D | D | D | D | ? | ? |
| FGSIS  (4 items) | Mohammed and Hassan (2014) | I | I | D | I | I | NR | ? | NE |
| GSIS | Zielinski et al., (2012) | I | I | I | D | D | NR | ? | ? |
| GAS | Bramwell and Morland (2009) | D | I | D | I | I | NR | ? | NE |
| MGSIS  (5 items) | Saffari et al., (2016) | I | I | D | I | I | I | ? | NE |
| MGSIS  (7 items) | Arruda et al., (2021) | D | D | D | D | D | NR | + | NE |
|  | Koçak et al., (2023) | I | I | I | I | D | NR | ? |  |

PROM: Patient-Reported Outcome Measure. FGSIS: Female Genital Self-Image Scale. GSIS: Genital Self-Image Scale. GAS: Genital Appearance Satisfaction. MGSIS: Male Genital Self-Image Scale. FGSIS-S: FGSIS Spanish version. VG: very good. D: doubtful. I: inadequate. +: sufficient results. ?: indeterminate results. NR: not reported. NE: not evaluated.

**Table S3**

*PROMs structural validity*

| **PROM** | **Author (year)** | **Statistical method** | **Outcome** | **Methodological quality** | **Rating** | **Rating PROM** |
| --- | --- | --- | --- | --- | --- | --- |
| FGSIS  (4 items) | Herbenick et al., (2011) | CFA | CFI = 1.00, IFI = 1.00, RMSEA = 0.05 | VG | + | + |
| FGSIS  (7 items) | Herbenick et al., (2011) | CFA | CFI = 0.932, IFI = 0.93, RMSEA = 0.15 | VG | - | ± |
|  | Herbenick and Reece (2010) | EFA (PCA) | 4.15 (eigenvalues) | D | + |  |
|  | DeMaria et al., (2012) | EFA (PCA) and CFA | RMSEA = 0.08, CFI = 0.98, NFI = 0.97 | VG | + |  |
|  | Ellibes Kaya et al., (2019) | EFA and CFA | CFI = 0.938, NFI = 0.928, RMSEA = 0.109 (90%CI 0.086 - 0.133), RMR = 0.051, GFI = 0.956 | VG | - |  |
|  | Arruda et al., (2023) | EFA (PCA) | CFI = 0.987, TLI = 0.981,  RMSEA = 0.046 (90%CI 0.025 - 0.046) | VG | + |  |
|  | Komon et al., (2022) | CFA | CFI = 0.98, TLI = 0.97,  RMSEA = 0.07 (90%CI 0.02 - 0.16), SRMR = 0.05 | A | + |  |
|  | Pakpour et al., (2014) | EFA (PCA) and CFA | Eigenvalue F1: 3.381, Eigenvalue F2: 1.060, CFI = 0.99, GFI = 0.99, NFI = 0.98, RMSEA = 0.060 (95% CI 0.008 - 0.079), SRMR = 0.032, PNFI = 0.32 | VG | + |  |
| FGSIS-S  (6 items) | Bartolomé et al., (2022) | EFA | Eigenvalue: 3.23 (EV = 74.9%) | D | + | + |
| GAS | Veale et al., (2014) | EFA (PCA) | 3 factors | I | - | NE |
|  | Bramwell and Morland (2009) | EFA (PCA) | 3 factors (eigenvalues 1: 3.96, eigenvalues 2: 1.53, eigenvalues 3: 1.2) | D | ? |  |
| GSIS | Zielinski et al., (2012) | EFA (PCA) | 4 factors (eigenvalue: 9) | D | + | + |
| MGSIS  (5 items) | Saffari et al., (2016) | EFA (PCA) and CFA | Eigenvalue 5-item: 3.76. RMSEA = 0.050 (0.001 – 0.110), CFI = 1.0, NFI = 0.99, SRMR = 0.021, PNFI = 0.64 | VG | + | + |
|  | Herbenick et al., (2013) | EFA (PCA) and CFA | 1 factor (IFI = 1, TLI = 1, RMSEA = 0.05) | D | + |  |
| MGSIS  (7 items) | Herbenick et al., (2013) | EFA (PCA) and CFA | 1 factor (CFI = 0.95, IFI = 0.95, RMSEA = 0.14) | D | + | + |
|  | Arruda et al., (2021) | EFA and CFA | CFI = 1.000, TLI = 1.002, RMSEA = 0.000 | VG | + |  |
|  | Koçak et al., (2023) | EFA (PCA) and CFA | Eigenvalues: 4.955, RMSEA = 0.076, GFI = 0.974, AGFI = 0.933, CFI = 0.989, NFI = 0.984, IFI = 0.989, PNFI = 0.515, PGFI = 0.383 | VG | + |  |
| IMGI | Davis et al., (2013) | EFA (PCA) | 6 factors | D | - | ± |
|  | Omar et al., (2016) | EFA (PCA) | 14 items, 4 factors (EV = 70.2%) | D | + |  |
| K-GIS | Lim and Cho (2018) | EFA and CFA | CFI = 0.912, RMSEA = 0.053 (0.050 - 0.057), TLI = 0.901 | VG | + | + |

PROM: Patient-Reported Outcome Measure. FGSIS: Female Genital Self-Image Scale. GSIS: Genital Self-Image Scale. GAS: Genital Appearance Satisfaction. MGSIS: Male Genital Self-Image Scale. FGSIS-S: FGSIS Spanish version. IMGI: Index of Male Genital Image. K-GIS: Korean Genital Image Scale. CFA: Confirmatory factor Analysis. CFI: Comparative Fit Index. EFA: Exploratory Factor Analysis. EV: Explained variance. GFI: Good ness-of-Fit Index. PCA: Principal component Analysis. RMR: Root-Mean-square Residual. RMSEA: Root Mean Square Error of Approximation. NFI: Normed Fit Index. AGFI: Adjusted Goodness of Fit Index. IFI: Incremental Fit Index. PGFI: Parsimony Goodness of Fit Index. TLI: Tucker-Lewis Index. PNFI: Parsimonious NFI. SRMR: Standardized Root Mean Square Residual. VG: very good. A: adequate. D: doubtful. I: inadequate. +: sufficient results. -: insufficient results. ?: indeterminate results. ±: inconsistent results. NE: not evaluated.

**Table S4**

*PROMs internal consistency*

| **PROM** | **Author** | **Statistical method** | **Outcome** | **Methodological quality** | **Rating** | **Rating PROM** |
| --- | --- | --- | --- | --- | --- | --- |
| FGSIS  (4 items) | Herbenick et al., (2011) | Cronbach’s α | 0.86 (4 item version) | VG | + | + |
|  | Mohammed and Hassan (2014) | Cronbach’s α | 0.89 to 0.93 | D | + |  |
| FGSIS  (7 items) | Herbenick et al., (2011) | Cronbach’s α | 0.91 (7 item version) | VG | + | + |
|  | Herbenick and Reece (2010) | Cronbach’s α | 0.88 | VG | + |  |
|  | DeMaria et al., (2012) | Cronbach’s α | 0.89 (total); 0.86 (factor 1); 0.82 (factor 2) | VG | + |  |
|  | Jansuwan et al., (2022) | Cronbach’s α | 0.80 | D | ? |  |
|  | Loewinski et al., (2022) | Cronbach’s α | 0.82 | D | ? |  |
|  | Ellibes Ellibes Kaya et al., (2019) | Cronbach’s α | 0.81 | D | + |  |
|  | Arruda et al., (2023) | Cronbach’s α | 0.82 | VG | + |  |
|  | Komon et al., (2022) | Cronbach’s α | 0.84 | D | ? |  |
|  | Pakpour et al., (2014) | Cronbach’s α | 0.86 (total); 0.83 (factor 1); 0.79 (factor 2) | VG | + |  |
| FGSIS-S  (6 items) | Bartolomé et al., (2022) | McDonald’s omega | 0.85 | VG | + | + |
| GAS | Veale et al., (2013) | Cronbach’s α | 0.78 (group labioplasty) and 0.84 (group control) | VG | - | ± |
|  | Bramwell and Morland (2009) | Cronbach’s α | 0.82 (total); 0.76 (factor 1); 0.73 (factor 2); 0.73 (factor 3) | VG | + |  |
| GSIS | Zielinski et al., (2012) | Cronbach’s α | 0.79 to 0.89 (subscales) | D | + | + |
| MGSIS  (5 items) | Saffari et al., (2016) | Cronbach’s α | 0.89 | VG | + | + |
|  | Herbenick et al., (2013) | Cronbach’s α | 0.92 (5 item version) | D | + |  |
| MGSIS  (7 items) | Herbenick et al., (2013) | Cronbach’s α | 0.93 (7 item version) | VG | + | + |
|  | Arruda et al., (2021) | Cronbach’s α | 0.90 | VG | + |  |
|  | Koçak et al., (2023) | Cronbach’s α | 0.92 | VG | + |  |
| IMGI | Davis et al., (2013) | Cronbach’s α | 0.89 | D | ? | - |
|  | Omar et al., (2016) | Cronbach’s α | 0.85 (total); 0.79 (factor 1); 0.77 (factor 2); 0.40 (factor 3); 0.71 (factor 6) | VG | - |  |
| K-GIS | Lim and Cho (2018) | Cronbach’s α | 0.92 | I | + | + |

PROM: Patient-Reported Outcome Measure. FGSIS: Female Genital Self-Image Scale. GSIS: Genital Self-Image Scale. GAS: Genital Appearance Satisfaction. MGSIS: Male Genital Self-Image Scale. FGSIS-S: FGSIS Spanish version. IMGI: Index of Male Genital Image. K-GIS: Korean Genital Image Scale. VG: very good. D: doubtful. I: inadequate. +: sufficient results. -: insufficient results. ?: indeterminate results. ±: inconsistent results.

**Table S5**

*PROM cross-cultural validity/measurement invariance*

| **PROM** | **Author** | **Statistical methods** | **Comparison Groups** | **Outcome** | **Methodological quality** | **Rating** | **Rating PROM** |
| --- | --- | --- | --- | --- | --- | --- | --- |
| MGSIS  (5 items) | Saffari et al., (2016) | MGCFA | Healthy men (n = 1297) *versus* men with erectile dysfunction (n = 467) | The first model (i.e., configural invariance) showed excellent goodness-of-fit indices (p = 0.091, RMSEA = 0.010 [0.0 - 0.028], CFI = 1.0, SRMR = 0.06, PNFI = 0.64). However, the more restrictive model (metric invariance) provided better goodness-of-fit indices (p = 0.112, RMSEA = 0.011 [0.001 - 0.024], CFI = 1.0, SRMR = 0.05, PNFI = 0.70) | VG | + | + |

PROM: Patient-reported Outcome measure. MGSIS: Male Genital Self-Image Scale. MGCFA: Multigroup Confirmatory Factor Analysis. RMSEA: Root Mean Square Error of Approximation. CFI: Comparative Fit Index. SRMR: Standardized Root Mean Square Residual. PNFI: Parsimonious Normed Fit Index. VG: very good. +: sufficient results.

**Table S6**

*PROMs test-retest reliability*

| **PROM** | **Author (year)** | **Test-retest time** | **Statistical method** | **Outcome** | **Methodological quality** | **Rating** | **Rating PROM** |
| --- | --- | --- | --- | --- | --- | --- | --- |
| FGSIS  (4 items) | Herbenick et al., (2011) | Approximately 2 weeks | Correlation | r = 0.78; p < 0.001 | D | + | NE |
|  | Mohammed and Hassan (2014) | 2 weeks | Pearson and paired t-test | 0.86 to 0.97; p < 0.001 | D | + |  |
| FGSIS  (7 items) | Jansuwan et al., (2022) | 2 weeks | ICC and Weighted kappa | 0.79 (95%CI 0.67 – 0.87); 0.27 to 0.72 | D | + | + |
|  | Ellibes Kaya et al., (2019) | 2 weeks | ICC | 0.95 (95%CI 0.89 – 0.97) | D | + |  |
|  | Arruda et al., (2023) | 10 to 14 days | ICC | 0.92 (95%CI 0.90 – 0.93) | VG | + |  |
|  | Komon et al., (2022) | 2 weeks | ICC | 0.93 | D | + |  |
|  | Pakpour et al., (2014) | 2 weeks | ICC | 0.86 (95%CI 0.81 – 0.89) (total); 0.81 (95%CI 0.76 – 0.85) (factor 1); 0.89 (95%CI 0.86 – 0.91) (factor 2) | D | + |  |
| FGSIS-S  (6 items) | Bartolomé et al., (2022) | 7 to 14 days | ICC | 0.86 | D | + | + |
| GSIS | Zielinski et al., (2012) | NR | Pearson | 0.88 | D | + | + |
| MGSIS  (5 items) | Saffari et al., (2016) | 15 days | ICC | 0.88 (95%CI 0.87 – 0.89) | D | + | NE |
|  | Herbenick et al., (2013) | Approximately 2 weeks | Correlation | r = 0.48; p < 0.001 (5 items) | D | - |  |
| MGSIS  (7 items) | Arruda et al., (2021) | 14 to 20 days | ICC | 0.80 (95%CI 0.75 – 0.84) | D | + | NE |
|  | Koçak et al., (2023) | 15 days | Correlation | r = 0.73; p < 0.001; p = 0.279 | D | + |  |

PROM: Patient-Reported Outcome Measure. FGSIS: Female Genital Self-Image Scale. GSIS: Genital Self-Image Scale. MGSIS: Male Genital Self-Image Scale. FGSIS-S: FGSIS Spanish version. ICC: Intraclass Correlation Coefficient. NE: not evaluated. NR: not reported. VG: very good. D: doubtful. +: sufficient results. -: insufficient results.

**Table S7**

*PROMs measurement error*

| **PROM** | **Author (year)** | **Statistical method** | **Outcome** | **Methodological quality** | **Rating** | **Rating PROM** |
| --- | --- | --- | --- | --- | --- | --- |
| FGSIS  (7 items) | Ellibes Kaya et al., (2019) | SEM_agreement_, SDC_ind_, SDC_group_, LoA, Bland and Altman graph | SEM_agreement_ = 0.28, SDC_ind_ = 0.78, SDC_group_ = 0.052, LoA = -0.213 to 2.818 | D | ? | ? |
|  | Arruda et al., (2023) | SEM, SDC, LoA, Bland and Altman graph | SEM = 1.469, SDC = 4.071, LoA = -4.359 to 3.788, Bland and Altman | VG | ? |  |
| GSIS | Zielinski et al., (2012) | Mean difference of the scores, standard deviation of the difference between the scores, SDC, LoA | Mean difference of the scores: 0.65; standard deviation of the difference between the scores: 1.8; SDC: 4.9 | D | ? | ? |
| MGSIS  (7 items) | Arruda et al., (2021) | SEM, SDC, LoA, Bland and Altman graph | SEM = 2.28, SDC = 6.30, LoA = -7.29 to 5.29, Bland and Altman | D | ? | NE |

PROM: Patient-Reported Outcome Measure. FGSIS: Female Genital Self-Image Scale. GSIS: Genital Self-Image Scale. MGSIS: Male Genital Self-Image Scale. SEM: Standard Error of Measurement. SDC: Smallest Detectable Change. LoA: Limits of Agreement. VG: very good. D: doubtful. ?: indeterminate results. NE: not evaluated.

**Table S8**

*PROMs hypothesis testing for construct validity – comparison of instruments*

| **PROM** | **Author (year)** | **Statistical method** | **Comparator instruments** | **Outcome** | **Methodological quality** | **Rating** | **Rating PROM** |
| --- | --- | --- | --- | --- | --- | --- | --- |
| FGSIS  (4 items) | Herbenick et al., (2011) | Pearson’s correlations | FSFI | Arousal (0.12), desire (0.20), lubricant (0.16), orgasm (0.14), pain (0.15), satisfaction (0.15), total (0.19) | VG | + | + |
|  | Mohammed and Hassan (2014) | Pearson’s correlations | FSFI | Desire (r = 0.815), arousal (r = 0.861), lubrication (r = 0.855), orgasm (r = 0.820), satisfaction (r = 0.832), pain (r = 0.884), total scale score (r = 0.872, p < 0.001) | VG | + |  |
| FGSIS  (7 items) | Herbenick and Reece (2010) | Correlations | FSFI | Desire (r = 0.03, p = 0.18), arousal (r = 0.18, p < 0.001), lubrication (r = 0.14, p < 0.001), orgasm (r = 0.17, p < 0.001), satisfaction (r = 0.19, p < 0.001), pain (r = 0.13, p < 0.001) and the total score (r = 0.20, p < 0.001) | VG | + | + |
|  | DeMaria et al., (2012) | Pearson’s correlations | BPSS-R | r = 0.337; p < 0.001 | VG | + |  |
|  | Jansuwan et al., (2022) | Pearson’s correlations | FSFI | Total (0.62, p < 0.05) | VG | + |  |
|  | Ellibes Kaya et al., (2019) | Pearson’s correlations | FSDS-R and FSFI | Total FSFI (r = 0.597)  FSDS-R (r = -4.51, p < 0.01) | VG | + |  |
|  | Arruda et al., (2023) | Pearson’s correlations | FSFI and BAS-2 | FSFI (0.372, p < 0.001)  BAS-2 (0.521, p < 0.001) | VG | + |  |
|  | Komon et al., (2022) | Pearson’s correlations | FSFI | r = 0.089 - 0.383, p < 0.05 | VG | + |  |
|  | Pakpour et al., (2014) | Pearson’s correlations | FSFI, BAS, and RSES | FSFI (r = 0.18 - 0.81, p < 0.05 for all)  BAS (r = 0.49, p < 0.001)  RSES (r = 0.41, p < 0.001) | VG | + |  |
| GAS | Veale et al., (2013) | Spearman’s correlations | COPS, COPS-L, HADS, DS-R, BIQLI, and PISQ | COPS (0.34, p < 0.05)  COPS-L (0.74, p < 0.01)  HADS-anxiety (0.29, p < 0.05)  HADS-depression (0.26, p > 0.05)  DS-R (0.26, p > 0.05)  BIQLI (-0.44, p < 0.01)  PISQ (0.22, p > 0.05) | A | + | + |
|  | Bramwell and Morland (2009) | Pearson’s correlations | ASI, BSS, and SES | ASI (0.28, p < 0.01)  BSS (0.30, p < 0.01)  SES (-0.41, p < 0.01) | VG | + |  |
| MGSIS  (5 items) | Saffari et al., (2016) | Pearson’s correlations | IIEF, BAS, and RSES | IIEF (r = 0.31–0.62, p < 0.01)  BAS (NS)  RSES (NS) | VG | + | + |
|  | Herbenick et al., (2013) | NR | IIEF | NR | I | + |  |
| MGSIS  (7 items) | Arruda et al., (2021) | Spearman’s correlations | IIEF, BAS-2, and RSES | IIEF (0.296, p < 0.001)  BAS-2 (0.458, p < 0.001)  RSES (0.350, p < 0.001) | VG | + | + |
| IMGI | Davis et al., (2013) | PCA | BASS | NR | I | + | + |
|  | Omar et al., (2016) | Pearson’s correlations | SHIM | r = 0.32–0.88, p < 0.05 | VG | + |  |
| K-GIS | Lim and Cho (2018) | Correlations | FGSIS, DSFI, and K-MSI | FGSIS (correlation > 0.217, p < 0.01)  DSFI (correlation > 0.258, p < 0.01)  K-MSI (correlation > 0.198, p < 0.01) | VG | + | + |

PROM: Patient-Reported Outcome Measure. FGSIS: Female Genital Self-Image Scale. GAS: Genital Appearance Satisfaction. MGSIS: Male Genital Self-Image Scale. IMGI: Index of Male Genital Image. K-GIS: Korean Genital Image Scale. FSFI: Female Sexual Function Index. BPSS-R: Body Parts Satisfaction Scale-Revised. FSDS-R: Female Sexual Distress Scale-Revised. COPS: Cosmetic Procedure Screening Scale. COPS-L: Cosmetic Procedure Screening Scale modified for labia. HADS: Hospital Anxiety and Depression Scale. DS-R: Disgust Scale Revised. BIQLI: Body Image Quality of Life Inventory. PISQ: The Prolapse–Urinary Incontinence Sexual Function Questionnaire. ASI: Appearance Schemas Inventory. BSS: Body Satisfaction Scale. SES: Self-Esteem Scale. BAS: Body Appreciation Scale. RSES: Rosenberg Self-Esteem Scale. IIEF: International Index of Erectile Function. BAS-2: Body Appreciation Scale. BASS: Body Areas Satisfaction Scale. SHIM: Sexual health inventory for men. DSFI: Derogatis Sexual Function Inventory. K-MSI: Korean-Marital Satisfaction Inventory. NR: not reported. VG: very good. A: adequate. I: inadequate. PCA: Principal Component Analysis. +: sufficient results. -: insufficient results.

**Table S9**

*PROMs hypothesis testing for construct validity – comparison between known groups*

| **PROM** | **Author (year)** | **Statistical method** | **Comparator groups** | **Outcome** | **Methodological quality** | **Rating** | **Rating PROM** |
| --- | --- | --- | --- | --- | --- | --- | --- |
| FGSIS  (4 items) | Herbenick et al., (2011) | t-test | I) Gynecological exam within the previous year; II) Genital self-examination within the previous month; III) Use of vibrator within the previous 30 days | I and II (p < 0.001)  III (p < 0.010) | D | + | NE |
| FGSIS  (7 items) | Herbenick and Reece (2010) | Bivariate correlations | I) Ever experienced orgasm during cunnilingus; II) Ever experienced orgasm during self-masturbation with a vibrator; III) Had a gynecological exam in the previous 12 months | Groups comparison: p < 0.001, omega squared = 0.01 | D | + | NE |
|  | DeMaria et al., (2012) | t-test | I) Had a gynecological exam in the previous 24 months; II) Number of vaginal intercourse partners during the past 3 months; III) Number of oral sex partners during the past 3 months | I (p = 0.01)  II e III (p < 0.01) | D | + |  |
|  | Ellibes Kaya et al., (2019) | Mann-Whitney U | Presence or absence of vaginal and/or clitoral masturbation | Absent (n = 360) Mean rank = 210.1, ≥ 1 / month (n = 101) Mean rank = 305.3 (p < 0.001) | D | + |  |
| FGSIS-S  (6 items) | Bartolomé et al., (2022) | Logistic regression | I) Have considered genital cosmetic surgery; II) Have concerns about the appearance of their genitals | Inverse relation. I (OR: 0.75, 95%IC 0.66 − 0.85; p < 0.001) and II (OR: 0.71, 95%IC 0.63 − 0.79; p < 0.001). | D | + | + |
| GSIS | Zielinski et al., (2012) | t-test and effect size | Have considered genital cosmetic surgery | p < 0.01 and p < 0.001. Effect size: GSIS-20 = 0.15 | D | + | + |
| GAS | Veale et al., (2013) | Mann-Whitney U | Labiaplasty and control groups | p < 0.001 | VG | + | + |
| MGSIS  (5 items) | Saffari et al., (2016) | ANOVA one -way | Age group, smoking status, and monthly family income | Age group (< 0.001), smoking status (< 0.001), and monthly family income (< 0.001) | VG | + | + |
| IMGI | Davis et al., (2013) | t-test | Sexual function, health variables, and psychosexual variables | Difficulty with erection, premature ejaculation, and were circumcised (p < 0.001); had an STI (p < 0.05) and marginally for men with no sexual experience (p = 0.07) | D | + | NE |

PROM: Patient-Reported Outcome Measure. FGSIS: Female Genital Self-Image Scale. GSIS: Genital Self-Image Scale. GAS: Genital Appearance Satisfaction. MGSIS: Male Genital Self-Image Scale. FGSIS-S: FGSIS Spanish version. IMGI: Index of Male Genital Image. OR: Odds Ratio. VG: very good. D: doubtful. +: sufficient results. NE: not evaluated.
